# East meets West: cross-cultural generalizability of the numinous motivation inventory in Poland and Vietnam

**DOI:** 10.3389/fpsyg.2026.1866025

**Published:** 2026-09-16

**Authors:** Ralph L. Piedmont, Klaudia T. Bochniarz, Jesse Fox, Thai Thi-Hong Bui, Huong Thu Tran, Joanna Różycka-Tran

**Affiliations:** 1 Center for Professional Studies, Timonium, MD, United States; 2 Institute of Psychology, Faculty of Social Sciences, University of Gdańsk, Gdańsk, Poland; 3 Department of Counselor Education, Stetson University, Deland, FL, United States; 4 Faculty of Psychology, University of Social Sciences and Humanities, Vietnam National University, Hanoi, Vietnam

**Keywords:** distress, emotional dysphoria, help-seeking behavior, numinous, religion, spiritual/religious coping

## Abstract

**Background:**

The motivations to find durability to our mortal existence (i.e., infinitude), coherence in the midst of otherwise chaotic experiences (i.e., meaning), and a sense that our lives are worthy in the unfolding story of our species (i.e., worthiness), are powerful psychological qualities that, in part, comprise the uniqueness of our humanity. The psychological dimension hypothesized to encapsulate these qualities is referred to as *The Numinous*.

**Methods:**

This study tested competing measurement models to ascertain how universal numinous motivations are across two culturally distinct people groups: Polish (*N* = 328) and Vietnamese (*N* = 307) citizens. In addition, it tested if these motivations help to explain psychological qualities including spiritual bypass, psychological dysphoria, and help-seeking over and above the Five Factor Model (FFM) of Personality.

**Results:**

The findings showed that despite cultural differences in the two samples, the Numinous remained structurally similar across both cultures, was discriminant from personality, and predicted psychological qualities independently from the FFM.

**Discussion:**

The findings supported the hypothesis that numinous motivations are a universal quality of human psychology, that they are independent of personality, and predict unique pathways of psychological distress and help-seeking strategies.

## 1 Introduction

Over the past 30 years the Five-Factor Model (FFM) of personality has emerged as the dominant taxonomic model of personality. The dimensions of Neuroticism, Extraversion, Openness, Agreeableness, and Conscientiousness have been demonstrated to be genetically based, cross-culturally generalizable, as well as predictively and interpretively powerful (Widiger, 2017). However, the emergence of the FFM has been accompanied by criticisms and reservations by some in the field. These critiques have centered on issues about the model’s lack of ontological and theoretical depth (e.g., whether it is truly a comprehensive model of personality dynamics) as well as whether the true complexity of personality can be completely reflected in only five factors (Loevinger, 1994; Block, 1995). This latter issue carries important implications for the ultimate utility of the model. While the FFM is based on lexigraphic analyses of the 17,956 adjectives originally identified by Allport and Odbert (1936), only small subsets of these adjectives have really been tested in research. Thus, critics have wondered whether the inclusion of more of these adjectives in research would generate new personality domains.

In a series of studies by Goldberg and colleagues (e.g., Ashton et al., 2004), these researchers explicitly included large numbers of adjective ratings scales that were more representative of the original set. When looking beyond five factors, other dimensions were found. Most notably, these authors discovered a sixth factor they labeled “Honest-Humility” which they found to be recoverable over multiple samples. This finding was the basis for the development of the HEXACO model. Interestingly, these same authors also found a replicable seventh factor labelled *Religiousness* (Ashton et al., 2004). When researchers expanded their adjective lists and extracted more than five factors, Religiousness was consistently recovered (Saucier and Goldberg, 1998). Ashton et al. (2004, p. 719) concluded, “Religiosity can now be added to the diverse array of seventh factors that have emerged, additional to the six widely replicated dimensions, within seven-factor solutions derived from personality-descriptive terms.”

While these authors noted that Religiousness was considered a personality domain in other models (e.g., Cloninger et al., 1993; Piedmont, 1999), they believed Religiosity to be different from the other FFM domains and thus fell outside of the domain of personality proper. Their reasons were that Religiousness is based on a system of beliefs and social attitudes and that it is influenced by environmental forces to a greater extent than is found for the FFM domains. This is a curious statement because a brief review of Allport and Odbert’s list of personality adjectives by the first author yielded over 80 religious/spiritual terms considered personality descriptors (e.g., faithful, soulful, beneficent, faithless, irreligious, and unbelieving). Nonetheless, Ashton et al. (2004, p.719) also noted “Most researchers would probably agree that Religiosity is a very important dimension of individual differences.”

However, Piedmont (1999, 2026) took a different perspective. He noted that religious/spiritual dynamics are a uniquely human quality that pervades all societies, across time and culture. These dynamics appear to exert a strong influence over the behavior of people and serves to direct the flow of both individuals and cultures. His work aimed to determine whether there was a more fundamental, uniquely human motivation that may be operating. His research identified the construct of Spiritual Transcendence and data supported the contention that this construct represented a new, independent personality dimension (Piedmont, 1999; see Piedmont and Wilkins, 2019 for a comprehensive overview of this program of research). Ultimately, Piedmont recognized that underlying spiritual/religious dynamics is a more fundamental dimension that is related to our ultimate existential drives, which he has termed *the Numinous.*

The Numinous works out of our tremendous cognitive capacities to think broadly, creatively, and complexly across long time periods. These capacities are the core adaptive qualities of our species, and also apply to self-examinations of personal worth, meaning, and durability. The Numinous takes these essential existential elements and combines them with the concept of psychological time perspective (PTP; Lewin, 1951) to create the Numinous. PTP reflects how people think about time in understanding their own lives; they can be past, present, future, or transcendentally oriented. Researchers developed a time perspective scale that captured these time orientations and noted that those with a future time orientation exhibited more successful lives than those with a past or present orientation (Boyd and Zimbardo, 1997; Zimbardo and Boyd, 1999). However, those who possessed a transcendental time perspective, a belief that one’s existence continues on after death, were the most resilient individuals who had a capacity to transform their lives into more socially engaged, prosocial styles.

Piedmont (2017) developed the Numinous Motivation Inventory (NMI) to capture this new dimension. The NMI assesses three dimensions that reflect the three major existential questions all people grapple with in their lives (Tillich, 2000): (1) is death the end? (2) does life carry an intrinsic meaning? And, (3) does my life matter and have worth? These three issues serve as the core dimensions of the Numinous: 1) *Infinitude*, examines the extent to which individuals understand their lives as being one step in an infinite ontological journey or not; 2) *Meaning,* the extent to which individuals can identify patterns and logical processes in life; and, 3) *Worthiness,* the ability to understand that despite our weaknesses and flaws, fundamentally our lives make an important and useful contribution to the world.

Research has shown that the NMI meets the empirical criteria for being considered a new, independent dimension of personality: it has been shown to be independent of the FFM domains, possesses significant incremental validity over the FFM, has multiple facets, has shown to be cross-culturally valid, and demonstrated causal precedence in predicting important psychological outcomes (e.g., emotional dysphoria, interpersonal style, well-being; see: Piedmont and Wilkins, 2019 for a listing of all seven empirical criteria).

While the Numinous model hypothesizes that these existential motivations are universal, it is clear that their expression varies across cultures. The important issue to determine is whether these differences reflect merely stylistic, culturally informed processes or if different cultures understand existential issues in very different ways. For example, Asian culture is much more collectivistic in its focus than Western cultures and consequently this difference may have a significant impact on how existential issues are understood. Piedmont et al. (2026) examined the NMI in a sample of Chinese nationals and found that the factor structure and mean level scores were identical to US normative data and that scores on the NMI significantly, and incrementally, predicted issues of emotional dysphoria and meaning over the domains of the FFM. The NMI was also found to be orthogonal to the FFM domains.

The purpose of this study was to add to the cultural generalizability of the NMI into two new cultures: Polish and Vietnamese. These two cultures are very different from one another and provide an opportunity to rigorously evaluate the robustness of the Numinous in different cultures. This study will examine four important issues: (1) Does the NMI remain independent of the FFM domains in both cultures? (2) Can the factor structure of the NMI be recovered intact in both contexts? (3) Does the NMI evidence significant incremental predictive validity over the FFM in a diverse range of outcomes (e.g., coping, help-seeking attitudes, and emotional dysphoria)? (4) Finally, Structural Equation Modeling (SEM) will be used to evaluate several causal models that examine how the NMI relates to these outcomes relative to the FFM domains. The model that postulates the NMI as a unique, incremental predictor is expected to be the best-fitting model. Confirmation of these hypotheses will provide strong empirical support for both the conceptual and empirical value of the Numinous. Such findings will demonstrate that the existential strivings of people represent a unified set of intrinsic qualities that provide the highest level of motivation, and adaptiveness, in our psychological system.

### 1.1 Cultural approaches to ultimate existential issues

In the present study, we focus on the Polish and Vietnamese contexts and samples. These two cultural contexts provide an interesting comparison of how the Numinous is expressed, as they differ in several important socio-cultural, philosophical, and religious characteristics; however, both countries shared somewhat similar political frameworks during the twentieth century.

#### 1.1.1 Polish cultural context to the numinous

For many years, being Catholic was part of Polish identity (Porter-Szucs, 2011). Currently, approximately 91% of the Polish population identifies as Catholic (Główny Urząd Statystyczny (GUS), 2025), although sociologists emphasize significant ongoing changes, including processes of secularization, declining religious practice, and the growing individualization of belief (Hackett et al., 2025). Catholics perceive life as a path toward salvation and a relationship with God. Suffering is interpreted as a test, purification, or participation in the suffering of Christ, while death is seen as a transition to eternal life. This afterlife is understood as continued existence in communion with God, often framed in terms of heaven, purgatory, or hell, depending on one’s moral and spiritual condition, which reflects a predominantly linear and teleological understanding of human existence, oriented toward an ultimate, transcendent end (Eliade, 1954). At the same time, Poland’s spiritual culture is deeply rooted not only in the influence of Catholicism but also in folk traditions and philosophical reflection. Folk traditions further enrich this framework by embedding religious meanings in everyday practices and seasonal rituals, often structured around the liturgical and agricultural calendar, in which Catholic celebrations incorporate elements of earlier folk beliefs (Krawczyk-Wasilewska, 2015). Moreover, encounters with the sacred are often expressed through art and literature, reflecting a sensitivity to the mysterious and transcendent aspects of life, similar to other individualistic cultures (Piedmont, 1999).

Furthermore, this existential orientation cannot be understood without reference to Poland’s historical experience. The country’s history, marked by periods of social and political destabilization, including the partitions, World War II, and decades under a communist regime, has made questions of meaning, suffering, and the balance between adaptation and individual autonomy particularly salient. These conditions fostered the development of psychological theories addressing existential struggle and personal transformation. One such concept is the theory of positive disintegration developed by Kazimierz Dąbrowski (1979), which describes personality development as a process involving the transcendence of primary ego structures. According to this theory, psychological disintegration resulting from internal conflicts and existential dilemmas is not merely destructive; it also enables the emergence of a more autonomous, reflective, and value-oriented personality. This perspective highlights the constructive role of existential tension in human development.

This line of thought was further elaborated by the Polish priest and psychologist Kazimierz Popielski, who developed the concept of the noetic dimension of personality within the framework of Viktor Frankl's (1963) logotherapy and existential analysis. Popielski contributed to the empirical and theoretical elaboration of noetic constructs (Popielski, 1994). Central to his theory was the meaning of life, which he sees as a fundamental motivational force guiding human actions, choices, and personal growth. In his view, human beings are not only biological and psychological but also possess a spiritual (noetic) dimension that shapes their search for meaning, which can be conceptualized as a distinct dimension of personality. He emphasizes the multidimensional nature of human existence, where physical, emotional, cognitive, and spiritual aspects interact, and the multidirectional nature of development, allowing for multiple paths rather than a single fixed trajectory (Popielski, 1994, 1999). For his contributions, he is regarded as the founder of logotherapy in Poland.

#### 1.1.2 Vietnamese cultural context of the numinous: meaning, worthiness, mortality, and infinity

In the Vietnamese cultural context, experiences of the Numinous are shaped by a long-standing synthesis of indigenous beliefs and the philosophical traditions of Buddhism, Confucianism, and Taoism (Taylor, 2007). Rather than emphasizing a strict separation between the sacred and the secular, Vietnamese typically understand the Numinous as immanent—embedded within the natural and social world. Sacred presence is often perceived in places such as village temples, communal houses, rivers, or ancient trees, reflecting an animistic worldview in which spiritual forces permeate everyday life (Anh, 2010; Cadière and Đỗ, 2015). As a result, the Numinous is not primarily defined as an individual relationship with a transcendent reality, but as a relational system connecting individuals with ancestors, community, and the broader cosmic order.

Meaning in life in the Vietnamese cultural framework is commonly interpreted through fulfilling social roles within the family and community, including filial piety toward parents, responsibility for raising children, and contributing to the continuity and reputation of the family lineage (Luong, 1992; Anh, 2010). In this sense, meaning emerges not only from personal purpose but from maintaining harmony with ancestors, descendants, and the community. Closely related to this orientation is the cultural understanding of worthiness. Rather than being primarily an internal evaluation of self-worth, worthiness in Vietnamese culture is often expressed through the successful fulfillment of ethical and relational duties. A person is considered worthy when they demonstrate filial devotion, moral integrity, and responsibility toward family and society.

These beliefs also shape distinctive cultural perspectives on mortality and infinity. In Vietnamese thought, death is generally not interpreted as a final termination of existence but as a transition between interconnected realms. The traditional expression “sinh ký tử quy” (“life is a temporary stay; death is a return”) reflects the belief that the deceased continue to exist in another dimension while maintaining influence over the fortunes of the living. Thus, immortality is culturally framed through social and genealogical continuity rather than purely individual transcendence (Phan, 1993). Together, these perspectives illustrate how Vietnamese integrate existential questions of meaning, worthiness, mortality, and continuity into a relational worldview grounded in family, community, and the ongoing interaction between the visible and invisible worlds.

#### 1.1.3 Cultural context: conclusions

These brief cultural overviews make apparent the reality of existential issues in both cultures. Issues of meaning, worthiness, and infinitude seem to be a common set of salient and relevant dynamics. What differs is the cultural orientation of individualism vs. collectivism. But despite the different perspectives each represents, the content of these existential engagements appear almost identical. Thus, there seems to be a common lexical platform in both cultures surrounding existential themes, which makes translating the NMI an appropriate endeavor. After controlling for these cultural variations, it seems likely that the constructs represented in the NMI will be clearly recovered and their unique, predictive abilities clearly evidenced.

### 1.2 Hypotheses and analytic plan

The first hypothesis (H1) regarding the NMI is that responses to the scales in both the Polish and Vietnamese samples would all have alpha reliabilities consistent with normative data. Further, United States normative information will be used to score the NMI responses by transforming raw scores into *T*-scores having a mean of 50 and a standard deviation (SD) of 10. This was being done for two reasons. First, using US norms would provide an interpretive framework for understanding the distributional characteristics of the Polish and Vietnamese raw data (see Cheung and Song, 1989). Do scores fall in the average range of US scores (e.g., have *T-*scores between 45 and 55)? Do they have a comparable level of dispersion (e.g., SDs between 9 and 12) as US scores? If so, that would indicate a common level of engagement in existential issues across these two cultures. Second, the normative data controls for US gender and age effects. Examining gender differences on these *T*-scores determines whether there are different or more extreme gender effects. While US and European data indicate women consistently scoring higher than men on the Numinous (e.g., Piedmont, 2017; Fox and Piedmont, 2020; Piotrowski et al., 2021) there is some evidence that in Asian samples, men may score higher than women (e.g., Piedmont and Leach, 2002; Piedmont et al., 2026) or there are no gender differences (e.g., Piedmont, 2007; Le et al., 2019; Tabaeian and Piedmont, 2024).

The second hypothesis (H2) is that the putative three-factor structure of the NMI will be recovered in these samples, thereby supporting the structural congruence and cross-cultural replicability of these constructs. Because the NMI factor structure is well established, Structural Equation Modeling (SEM) will be used to determine whether the putative three correlated factor structure can be recovered in both samples.

The third hypothesis (H3) is that in each sample, the three-factor correlated structure of the scale will be compared to the US normative structure, with the expectation that the loadings will be identical. While SEM is an apt method for determining whether a particular factor structure can be recovered in a specific sample, we believe that it is not the best method for determining whether or not factor structures in two different samples are identical, especially when these samples represent distinct cultures (O’Connor, 2017).

In SEM, the covariances among a set of items are analyzed and a linear set of weights is derived to maximize their inter-relatedness. In cross-cultural comparisons, these sets of weights (usually normative values in the original culture versus those obtained in the new culture) are directly compared and their similarity determined by a nonparametric Chi-square test. If the two sets of weights match, then the chi-square test statistic is found to be nonsignificant, indicating that the scale items overlap identically in the two cultures. Conversely, if the two weight sets do not match, SEM cannot determine the reason for the lack of fit, as the chi-square only tests whether the observed numbers match expected values without making further assumptions or inferences about their nature.

There are two primary explanations for a lack of congruence between factor structures. The most direct is that the scale’s items function differently, or are understood differently, in the cultures being compared. Alternatively, and central to the aims of cross-cultural research, is the possibility that culturally-based stylistic factors are influencing the interrelatedness of the items. These stylistic processes, while of potential conceptual value, introduce systematic error that needs to be accounted for prior to examining congruence between sets of weights. Confirmatory factor analysis (CFA) is particularly well-suited to addressing this type of issue.

Factor analysis provides a different way of understanding inter-scale relationships by representing them spatially (the correlation between two variables is rendered as the cosine of the angle between the two variables). Once variables are situated, interpretive axes are overlayed in order to better understand/identify groupings of similar items. The factor loadings are the relationships between each item and its defining axis. When factor analyzing the same items in two different samples in order to compare their loadings, it is important to make sure that the placement of the interpretive axes in the new sample has a similar location as those in the original sample. This type of “targeted rotation” corrects for any sample specific qualities (e.g., cultural styles, error) that may be distorting the location of the axes in the new sample. Once the axes placements are in alignment with the original factor solution, then loadings can be directly compared. Culturally-based stylistic factors can and do impact factor analysis results.

For example, Piedmont and Chae (1997) examined the factor structure of the Korean language NEO PI-R. When the scale was factored, the domains of N, O, and C were recovered intact, although the E and A domains were found to intermingle. E and A are the anchor domains for interpersonal style. In United States samples, the placement of these factors evidences a Love vs. Power orientation. In the Korean sample, the pattern of loadings represented more of a Surgency vs. Benevolence orientation. This result had also been found in other Asian samples (e.g., Katigbak et al., 1996). Rather than indicating a problem with construct validity, these findings showed that these constructs, in more communal societies, take on a different expression. A 15– to 20-degree shift in the axes brings the factor analysis results into perfect alignment with United States normative structure. SEM is unable to evaluate whether the lack of fit for a structure is due to invalidity or rotational variability (Hopwood and Donnellan, 2010).

McCrae et al. (1996) proposed an efficient targeted rotation process for determining the comparability of factor structures. For the purposes of this study, their method involves conducting a conventional principal components analysis on the cross-cultural data sets and then submitting the results to an orthogonal Procrustes rotation (Schönemann, 1966) that uses NMI normative factor loadings (Piedmont, 2017, p.21) as the target matrix. Congruence coefficients (Gorsuch, 1983) are then calculated to assess the degree of fit for the two factor structures. Significance levels can be readily determined through Monte Carlo analyses that fit random data to the normative values (the NMI manual provides these values in Appendix E). This method has proven useful for assessing structural congruence and cross-cultural replicability of the NMI factor structure (e.g., Piedmont et al., 2020, 2026). Van de Vijver and Leung (1997) have also recommended this technique for studies evaluating the generalizability of a scale to a new cultural context.

The fourth hypothesis (H4) is that the NMI scales capture aspects of the individual that are independent of the domains of the Five-Factor Model (FFM) of personality. Piedmont (2017) demonstrated that the NMI scales were orthogonal to the FFM domains and demonstrated incremental predictive validity over them (see also Piedmont et al., 2020, 2023). To examine the incremental predictive validity of the NMI over the FFM, partial correlations between the NMI and the set of outcome measures (i.e., coping ability, affective distress, treatment-seeking attitudes, and spiritual bypass), controlling for the FFM will demonstrate significant, theoretically appropriate, replicated associations with these constructs. Specifically, NMI Worthiness ought to predict symptom scores independently of the FFM. The NMI will also uniquely correlate with coping scales related to spiritual and religious constructs and will also relate to treatment-seeking attitudes for physical and mental health problems.

The fifth hypothesis (H5) will explore the ontological role of the NMI in these associations. Because the Numinous is seen as an independent dimension of personality (Piedmont, 2026), it needs to be demonstrated that its motivational nature impacts psychological functioning and is not merely an outcome of other psychological processes. The NMI facet of Worthiness has been demonstrated to be uniquely predictive of emotional dysphoria (Piedmont et al., 2020)), it has also been shown in a longitudinal sample of undergraduate business majors to be a potential causal predictor of multiple aspects of emotional well-being (e.g., Flourishing, cognitive flexibility, and scores on the Depression, Anxiety, and Stress Scale; Piedmont et al., 2023). SEM will be used to evaluate the relevance of three different predictive models that vary the role of Worthiness as either a unique predictor of emotional distress or as an outcome of personality and affective dysphoria (see Figure 1). It is expected that the model that describes Worthiness as an incremental predictor of distress over the FFM will emerge as the model that best fits the data, supporting the hypothesized causal nature of the Numinous on psychological functioning.

**Figure 1:**
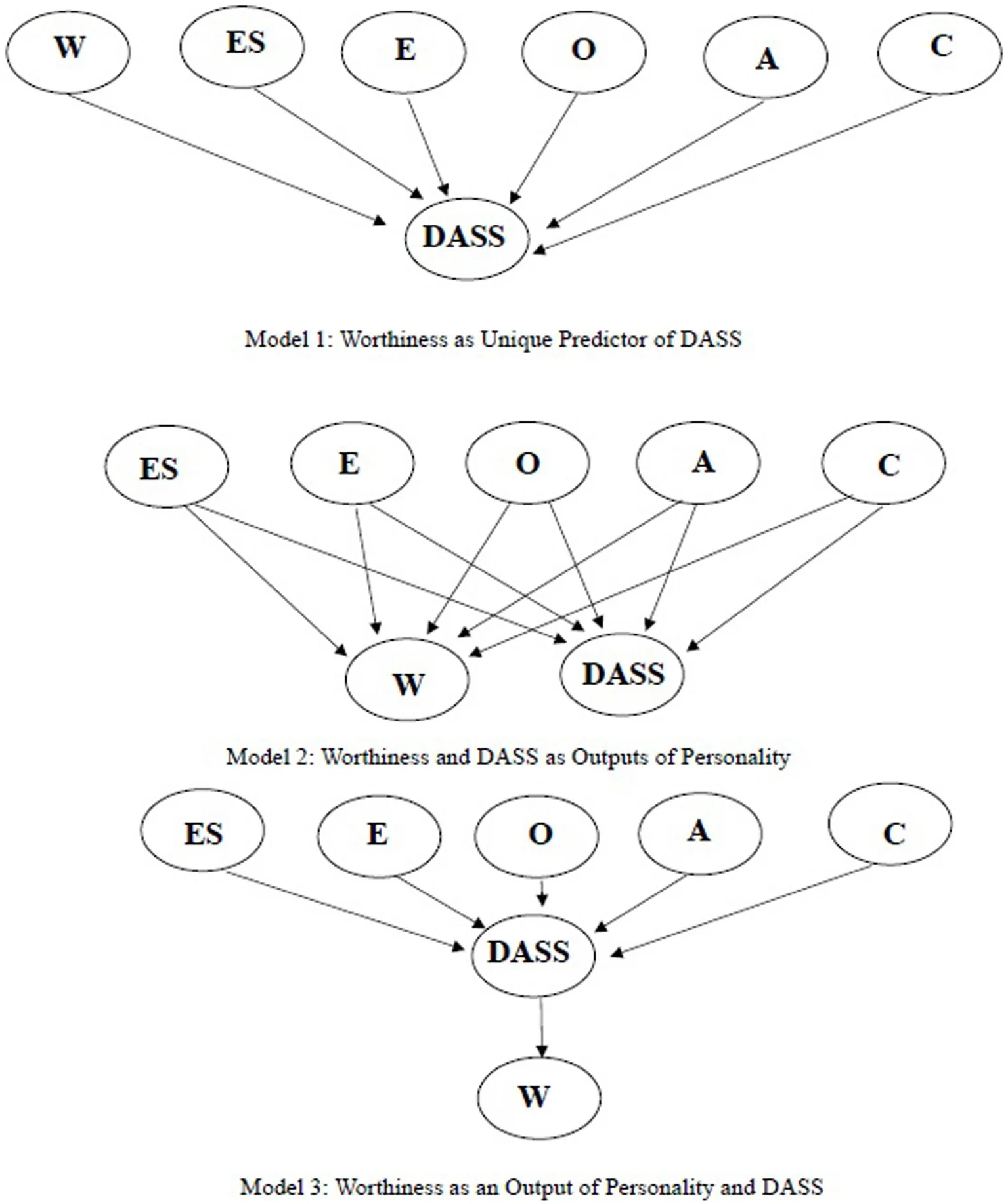
Proposed structural models linking personality, NMI Worthiness, and affective dysphoria. *W*: NMI Worthiness; *ES*: Emotional Stability; *E*: Extraversion; *O* = 1: Intellect; *A*: Agreeableness; *C* = 1: Conscientiousness; DASS: Depression, Anxiety, Stress Scale.

## 2 Methods

### 2.1 Participants

To ensure data quality and the relevance of the analytical sample, a series of exclusion criteria was applied to responses collected from both samples. First, responses from participants with excessively short completion times were removed, as very short response times may indicate insufficient effort or careless responding (Meade and Craig, 2012). This threshold corresponded to the minimum time required to read the instructions and provide thoughtful responses, as established during pilot testing. Second, participants who provided incorrect responses to attention-check items (e.g., instructions such as “please select response option 2”) were also excluded.

#### 2.1.1 Polish sample

This group consisted of 249 women and 79 men ranging in age from 18 to 68 (M = 29.50, SD = 12.34). Forty-two percent were single, 53% were in a committed relationship, 1% were widowed, and 3% were divorced. On average, this sample had 14 years of education (range: 10–36 years). Concerning religious affiliation, 50% were Catholic, 42% were Atheist/Agnostic, and the remaining 8% were either other Christian, Other Faith, or Buddhist.

#### 2.1.2 Vietnamese sample

This group consisted of 243 women and 64 men with a mean age of 20.50 years (*SD* = 4.50). Ninety-four percent were single, 5% were in a committed relationship and less than 1% were divorced. On average, this sample had 14 years of education (range: 10–26). Concerning religious affiliation, 43% were Atheist/Agnostic, 28% were Buddhist, 9% were Catholic and the remaining 20% were spread across other faith traditions, including Protestantism, Orthodox, and Islam.

### 2.2 Measures

#### 2.2.1 The depression, anxiety, and stress Scale-21 (DASS-21)

Developed by Lovibond and Lovibond (1995), the DASS is a 21-item scale used to measure symptoms common to psychological distress: depression (“I felt that I had nothing to look forward to”), anxiety (“I was aware of dryness of my mouth”), and stress (“I felt that I was using a lot of nervous energy”). In the present study, the Polish adaptation of the scale by Makara-Studzińska et al. (2022) and the Vietnamese adaptation by Le et al. (2017) were administered. Participants read each item describing a symptom and indicated the extent to which it applied to them over the past week using a four-point Likert scale ranging from 0 (*Did not apply to me at all*) to 3 (*Applied to me very much or most of the time*). The scale has shown strong psychometric properties across diverse cultural contexts (Le et al., 2025). Alpha coefficients for the current study are reported in Table 1.

**Table 1:** Descriptive statistics, gender differences, and alpha reliabilities for outcome measures in both the Polish and Vietnamese samples.

Scale	Men		Women		*t*-diff	α
	Mean	SD	Mean	SD		
Polish Sample^A^						
*DASS*						
Stress	7.25	5.82	9.75	5.91	−3.28***	0.91
Anxiety	4.70	5.20	6.24	6.49	−2.14*	0.88
Depression	5.84	5.76	6.37	5.54	−0.73	0.91
Total Score	17.78	15.68	22.35	15.61	−2.26***	0.95
*Coping*						
Problem Focused	1.83	0.60	1.96	0.56	−1.83	0.82
Emotional Focused	1.36	0.43	1.45	0.38	−1.67	0.58
Avoidant Coping	0.73	0.54	0.92	0.47	−2.97**	0.73
Religious Coping	0.57	0.88	0.73	0.93	−1.37	0.91
*Spiritual Bypass*						
Psychological Avoidance	28.82	8.04	27.72	7.57	1.11	0.95
Spiritualizing	14.13	3.15	13.89	3.04	0.59	0.93
*Help Seeking*						
Medical Help	1.78	0.62	2.29	0.51	−7.33***	0.83
Psychological Help	2.40	0.60	2.09	0.70	3.54***	0.72
Vietnamese Sample^B^						
*DASS*						
Stress	9.65	4.52	10.53	4.70	−1.34	0.85
Anxiety	7.45	4.17	8.30	4.34	−1.40	0.80
Depression	6.50	4.72	6.43	4.66	0.10	0.87
Total Score	23.61	11.97	25.26	12.30	−0.96	0.93
*Coping*						
Problem Focused	1.97	0.55	2.00	0.54	−0.29	0.81
Emotional Focused	1.62	0.46	1.64	0.48	−0.28	0.71
Avoidant Coping	0.84	0.44	0.98	0.46	−2.23*	0.67
Religious Coping	1.03	1.01	1.06	0.90	−0.19	0.80
*Spiritual bypass*						
Psychological Avoidance	26.33	5.77	26.24	5.07	0.12	0.86
Spiritualizing	13.03	2.71	12.93	2.63	0.18	0.85
*Help Seeking*						
Medical Help	1.62	0.45	1.68	0.43	−0.95	0.63
Psychological Help	2.26	0.55	2.17	0.63	1.03	0.69

^A^*N* Women = 249; *N* Men = 79; ^B^ *N* Women = 243; *N* Men = 64.

*p < 0.05. **p < 0.01. ***p < 0.001, two-tailed.

#### 2.2.2 International personality item Pool big five (IPIP-20)

Personality traits were assessed using the Polish version of the IPIP, the IPIP-BFM-20 (Topolewska et al., 2014). This 20-item instrument measures five major personality dimensions: Extraversion (E; e.g., “*I am the life of the party*“), Agreeableness (A; e.g., “*I sympathize with others’ feelings*”), Conscientiousness (C; e.g., “I follow a schedule”), Emotional Stability (ES; e.g., “I have frequent mood swings”), and Intellect (O; Openness to Experience; e.g., “I am full of ideas”), with four items assessing each trait. Participants indicated the extent to which each statement described them using a five-point Likert scale ranging from 1 (*very inaccurate*) to 5 (*very accurate*). Previous research has shown that the instrument demonstrates very good reliability (Topolewska et al., 2014).

In the Vietnamese context, no formally validated adaptation of the IPIP-BFM-20 is currently available. However, official Vietnamese translations of IPIP items are accessible through the International Personality Item Pool website (translated by Kate E. Walton). For the present study, the Vietnamese translations corresponding to the 20 items included in the Polish IPIP-BFM-20 were selected from this source. Subsequently, these translations were reviewed by independent Vietnamese bilingual experts to ensure linguistic clarity, cultural appropriateness, and conceptual equivalence with the original items. Alpha coefficients for the ES, E, O, A, and C scales in Poland were: 0.80, 0.84, 0.65, 0.62, and 0.77, respectively. For the Vietnamese sample, alphas were: 0.60, 0.49, 0.55, 0.60, and 0.50, respectively.

#### 2.2.3 Numinous motivation inventory (NMI)

Developed by Piedmont (2017), this 22-item scale captures one’s Numinous orientation, which represents the need to address ultimate existential issues centering around three questions: (1) is death the end? (2) does life carry any meaning? And, (3) am I a worthy individual? The NMI contains three facet scales that address each of these issues: *Infinitude* (e.g., “I believe that this life is only one stage in a larger, eternal process”); *Meaning* (“I am guided by my personal philosophy and/or faith”); and *Worthiness* (“I value myself”). Items are responded to on a 1 (*strongly disagree)* to 5 (*strongly agree)* Likert-type scale. Items are counterbalanced to control for acquiescence effects. The NMI has normative data that controls for age and gender. The NMI has been found to be a significant, unique predictor of psychological distress and characterological impairment in both theists and atheists (Piedmont et al., 2020), coping levels among physicians (Williamson et al., 2024), and existential growth among college business majors (Piedmont et al., 2023). Descriptive statistics and alpha reliabilities for scores on the NMI in both samples are presented in Table 2.

**Table 2:** Descriptive statistics, alpha reliabilities, and gender differences on the NMI scale by country.

NMI Scale	Men		Women		*t*-diff	Overall α
	M	SD	M	SD		
*Poland: Men n = 79 Women n = 249*						
Infinitude^A^	47.4	9.67	46.39	10.52	0.76	0.92
Worthiness	48.22	12.02	48.56	11.58	−0.23	0.92
Meaning	45.9	8.52	47.18	8.15	−1.2	0.71
Total Score	46.52	9.3	46.61	9.78	−0.07	0.89
*Vietnam: Men n = 64 Women n = 243*						
Infinitude^A^	51.42	7.32	49.58	7.1	1.83	0.79
Worthiness	50.24	10.75	47.36	10.59	1.93	0.92
Meaning	52.55	7.61	52.1	6.29	0.49	0.63
Total Score	51.64	8.6	49.12	7.93	2.23*	0.87

^A^Scores are presented as T-scores with a mean of 50 and SD of 10 based on US Normative Values (Piedmont, 2017).

*p < 0.05. **p < 0.01. ***p < 0.001, two-tailed.

#### 2.2.4 The spiritual bypass Scale-13 (SBS-13)

Spiritual bypass, a tendency to use spirituality as a means of avoiding psychological dysphoria, was assessed using the SBS-13 developed by Fox et al. (2017). This measure included 13 items rated on a 4-point Likert-type scale ranging from 1 (*strongly disagree*) to 4 (*strongly agree*). The items are grouped into two sub-facets. The first subscale, *psychological avoidance*, includes nine items (e.g., *I believe it is preferable to cure emotional problems by being spiritually advanced*). Higher scores on psychological avoidance indicate a tendency to avoid difficult emotions, experiences, or circumstances. The second subscale, *spiritualizing*, includes four items (e.g., *When someone I know is in trouble, I believe it is because they have done something wrong spiritually*). Higher scores on spiritualizing indicate a tendency to appraise ordinary scenarios and exaggerate their spiritual meaning. Prior studies have investigated construct validity of SBS-13, predictive and incremental validity over the Big Five, internal reliability, test–retest reliability, as well as language translation into Brazilian, Portuguese, Spanish, and Korean (Fox et al., 2017; Picciotto et al., 2018; Taylor et al., 2021; Ahmad et al., 2023a, 2023b).

#### 2.2.5 Attitudes toward seeking professional psychological help scale (ATSPPH)

Attitudes toward seeking professional psychological help were assessed using the short form of the ATSPPH, originally developed by Fischer and Farina (1995). The short form consists of 10 items (e.g., “Talking about problems with a psychologist seems to me as a poor way to get rid of emotional problems”) rated on a four-point scale ranging from 1 (*disagree*) to 4 (*agree*), with several items reverse-coded. Higher scores indicate more positive attitudes toward seeking professional psychological help. In this study, we used the Polish adaptation by Perenc and Radochonski (2016) and the Vietnamese version made by Ngo and Huynh (2009). The instrument has demonstrated good psychometric properties in previous studies (Picco et al., 2016). Reliability coefficients for these scales are presented in Table 1.

#### 2.2.6 Attitudes toward seeking medical help scale (ATSMHS)

The original version of the ATSMHS (Fischer et al., 2013) consists of 12 items (e.g., “When I have doubts or questions about my physical health, I find out what is wrong from a medical professional”) with answers scored on a four-point scale, ranging from 0 (*disagree*) to 3 (*agree*). However, no validated Polish or Vietnamese language adaptations of the full scale are currently available. Taking this into account, as well as the overall length of the questionnaire battery used in the present study, a shortened version of the scale was employed. Specifically, five items (items 3, 4, 6, 8, and 11 from the original version) were selected for inclusion. The selection of these items was based on their strong factor loadings on the factor reported in previous validation studies (Fischer et al., 2013). The instrument has demonstrated good psychometric properties in previous studies (Fox et al., 2020). Reliability coefficients for these scales are presented in Table 1.

#### 2.2.7 Brief COPE inventory

Developed by Carver (1997), the Brief COPE Inventory is a multidimensional self-report measure designed to assess a range of cognitive and behavioral coping responses individuals use when dealing with stress. The original version consists of 28 items, describing 14 distinct coping strategies, with two items assigned to each strategy. These strategies are organized into three higher-order coping domains: (I) Problem-focused coping, (II) Emotion-focused coping, and (III) Avoidant coping. In addition to the three overarching coping dimensions, the Brief COPE permits the calculation of specific facet-level subscales nested within these broader dimensions. In the present study, we used this scoring option to calculate Religious Coping as a separate two-item facet of the broader Emotion-Focused Coping dimension. Participants rated how frequently they engaged in each coping behavior using a four-point scale ranging from 0 (*I almost never do this*) to 3 (*I almost always do this*), with higher scores indicating more frequent use of a given coping strategy. The Polish adaptation used in this study was made by Juczyński and Ogińska-Bulik (2009), and the Vietnamese adaptation of the Brief COPE was made by Matsumoto et al. (2020). Reliability coefficients for these scales are presented in Table 1.

### 2.3 Procedure

#### 2.3.1 Translation process

The NMI, the SBS-13 and the ATSMHS needed to be translated into both Polish and Vietnamese. A commonly used and accepted multistep process involving translating and back-translating the scale was implemented (e.g., Hambleton, 1993). Initially, one of the research team members from Poland and one from Vietnam translated the items for these scales into the new language. These versions were then back-translated into English by two other, bilingual team members who were familiar with the psychological constructs. The first author (RLP) then examined these back-translated items with their original English. Items that were not clear or did not satisfactorily capture the constructs being assessed were identified, and, new translations were made. These changes were then sent to the other two bilingual team members for retranslation back into English. This process continued until the translations were deemed appropriate. At this point, the translated versions of the scale were deemed ready for administration.

#### 2.3.2 Research process

This study was conducted in accordance with the Declaration of Helsinki, and approved by the Research Ethics Committee at the University of Gdańsk, approval no. 31/2025/WNS. Informed consent was obtained from all subjects involved in the study. Data were collected using the online Profitest platform. Participants were informed about the purpose of the study and their right to withdraw at any time without penalty. Participants were not offered any incentives or financial compensation for completing the study. A non-probability convenience sampling strategy was used. The Polish sample was recruited from universities in the Tri-City area. Students enrolled in both full-time and part-time programs were invited to participate in the study. The Vietnamese sample was recruited from students attending major universities in Hanoi and Ho Chi Minh City. Vietnamese participants were asked to complete the survey during scheduled university classes.

## 3 Results

### 3.1 Descriptive statistics

Table 1 presents the descriptive statistics, alpha reliabilities, and gender effects for the outcome variables. Alphas for scores are similar in the two samples, indicating that scores on these constructs are equally reliable across these two different cultures. Mean level scores are also relatively similar, although there appears to be more gender differences in the Polish sample. The only replicated gender effect is for women using more Avoidant Coping strategies than men. In the Polish sample, with the exception of seeking psychological help, women scored significantly higher on psychological symptoms and taking a Avoidant Coping approach to dealing with stress.

Table 2 presents descriptive statistics and reliability coefficients for the NMI scales in both samples. When normative data are used to score the NMI, mean level scores are comparable to scores found in the US in both samples. However, overall means for the Vietnamese sample evidence significantly higher mean levels than the Polish sample on Infinitude [46.63 vs. 49.96, *t*(633) = −4.69, *p* < 0.001]. Meaning [46.87 vs. 52.19, *t*(633) = −8.95, *p* < 0.001] and overall Total NMI score [46.59 vs. 49.64, *t*(633) = −4.31, *p* < 0.001]. Concerning gender differences, only one effect emerged, in the Vietnamese sample, with men scoring significantly higher on the Total NMI score, *t*(305) = 2.23, *p* < 0.05. Alpha reliabilities for all scales across both samples are acceptable and values are comparable (Polish Mean *r_XX_* = 0.86; Vietnamese Mean *r_XX_* = 0.80).

### 3.2 Structural analyses

Separately for each sample, the underlying latent dimensionality of the NMI was evaluated using Structural Equation Modeling (LISREL 12.5.1). SEM was used to test for the presence of a 3-factor correlated model. For each analysis, the model was optimized based on the modification indices. Where indicated, correlated errors were fixed only among items on the same factor.

For the Polish sample, the final model presented a reasonable fit for the putative structure: chi square (*df* = 191) = 447.90, *p* < 0.001; chi square/df = 2.34, Root Mean Square Error of Approximation (RMSEA) = 0.06, Standardized Root Mean Residual (SRMR) = 0.08, Comparative Fit Index (CFI) = 0.94, Incremental Fit Index (IFI) = 0.94. According to Kline (2016), these results indicate acceptable fit. All items loaded significantly on their intended factor.

For the Vietnamese sample, a reasonable fit was also obtained: chi-square (*df* = 184) = 536.94, *p* < 0.001; chi-square/*df* = 2.86, RMSEA = 0.07, SRMR = 0.11, CFI = 0.90, IFI = 0.90. The values indicate acceptable fit for the 3-factor correlated structure. All items loaded significantly on their intended factor.

### 3.3 Structural equivalence with US norms

Separately for each sample, a principal components analysis was conducted and three oblique factors were extracted. These pattern loadings were then subjected to an orthogonal Procrustean Rotation that used the United States normative factor structure as the target matrix. Congruence coefficients (Gorsuch, 1983) were then calculated for each item, factor, and overall fit to determine the extent to which the recovered structure was identical to normative data. Each sample will be discussed in turn.

Table 3 presents the results for the Polish sample. In performing the initial principal components analysis, the KMO Sampling Adequacy coefficient was 0.91 indicating that the data were factorable and the Bartlett’s Test of Sphericity was Chi Square (*df* = 231) = 4637.29, *p* < 0.001 indicating the presence of significant overlap among the items. The scree plot clearly indicated that three factors were appropriate for extraction (first four eigenvalues were: 6.93, 4.86, 1.60, 0.96. As can be seen in Tables 3, 20 of the 22 items replicated their normative values and all three factors were clearly recovered, with congruence coefficients all above 0.95. The Polish sample clearly understood the NMI items similarly to US respondents. The observed factor structure can be considered identical to US norms.

**Table 3:** Principal components analysis using an orthogonal procrustean rotation of NMI items in Polish sample.

NMI Item	Procrustean Factor Loadings			Item Congruence
	Factor 1	Factor 2	Factor 3	
I1	**0.73**	0.02	−0.06	1.00***
I2	**0.85**	0.01	0.06	1.00***
I3	**0.76**	0.08	−0.13	0.99**
I4	**0.83**	0.03	0.05	0.99**
I5	**0.84**	−0.05	0.17	1.00***
I6	**0.72**	0.03	0.26	0.98*
I7	**0.76**	−0.05	0.28	1.00***
I8	**0.73**	−0.08	0.24	0.99***
W1	0.21	**0.54**	−0.23	0.95*
W2	0.03	**0.7**	−0.15	0.97*
W3	0.01	**0.78**	−0.04	1.00***
W4	0.06	**0.78**	−0.04	0.99**
W5	0.09	**0.78**	−0.02	1.00***
W6	−0.07	**0.86**	0.09	0.99**
W7	−0.14	**0.75**	0.12	0.99***
W8	−0.04	**0.89**	0.07	0.99***
W9	−0.12	**0.81**	0.26	1.00***
M1	0.21	0.2	**0.51**	0.89
M2	−0.09	−0.25	**0.75**	0.93*
M3	0.1	−0.22	**0.72**	0.99**
M4	0.15	0.22	**0.48**	0.93
M5	−0.03	0.04	**0.66**	0.98**
Factor Congruences	0.98***	0.98***	0.95***	0.98***

I = Infinitude. W = Worthiness. M = Meaning. *N* = 328.

Coefficients greater than 0.40 are given in bold.

*Congruence coefficient greater than 95% of rotations from random data.

**Congruence coefficient greater than 99% of rotations from random data.

***Congruence coefficient greater than 99.9% of rotations from random data (Piedmont, 2017).

In conducting the principal components analysis for the Vietnamese sample, the KMO Measure of Sampling Adequacy was 0.88 and Bartlett’s Test of Sphericity was: chi square (*df* = 231) = 3411.88, *p* < 0.001; again, indicating that there was sufficient overlap among the items to warrant a factor analysis. Scree analysis indicated that only three factors be extracted (first five eigenvalues were: 6.98, 6.38, 1.60, 1.10, 1.01). The resulting three oblique factors were subjected to an orthogonal Procrustean rotation that used US normative values as the target matrix and congruence coefficients determined how well the data fit; the results are presented in Table 4. As can be seen, 18 of the 22 items were significantly recovered and all three factors were also found to be identical with US normative factor structure. Congruence coefficients for each of the NMI factors and the overall coefficient clearly indicate that the observed factor structure is clearly equivalent to the US normative structure.

**Table 4:** Principal components analysis using an orthogonal Procrustean rotation of NMI items in a Vietnamese sample.

NMI Item	Procrustean Factor Loadings			Item Congruence
	Factor 1	Factor 2	Factor 3	
I1	**0.68**	0	−0.2	0.99***
I2	**0.61**	0.33	−0.24	0.86
I3	**0.34**	0.5	−0.2	0.7
I4	**0.74**	−0.01	−0.19	0.98*
I5	**0.69**	−0.22	0.29	0.96*
I6	**0.48**	0.1	0.34	0.88
I7	**0.56**	0.06	**0.44**	0.93**
I8	**0.45**	−0.2	**0.47**	0.93*
W1	0.19	**0.68**	−0.04	0.99**
W2	0	**0.83**	0.01	1.00***
W3	0.14	**0.77**	−0.05	1.00***
W4	0.15	**0.75**	−0.08	0.96*
W5	0.07	**0.87**	0.02	0.99**
W6	−0.01	**0.8**	−0.02	1.00***
W7	0.02	**0.65**	0.08	1.00***
W8	0.06	**0.86**	0.01	1.00***
W9	0.1	**0.77**	0.18	0.96**
M1	0.14	0.11	**0.67**	0.97*
M2	0.05	−0.28	**0.47**	0.84
M3	−0.06	0.01	**0.68**	0.97*
M4	0.04	0.11	**0.67**	1.00***
M5	0	0.11	**0.49**	0.98**
Factor Congruences	0.96***	0.96***	0.93***	0.95***

I = Infinitude. W = Worthiness. M = Meaning. *N* = 307.

Coefficients greater than 0.40 are given in bold.

*Congruence coefficient greater than 95% of rotations from random data.

**Congruence coefficient greater than 99% of rotations from random data.

***Congruence coefficient greater than 99.9% of rotations from random data (Piedmont, 2017).

### 3.4 External predictors

To examine the unique predictiveness of the NMI scales, partial correlation analyses were performed where NMI scales were correlated to the outcome variables controlling for the Big Five personality scores. These fifth-order partial correlations are presented in Tables 5 for the Polish sample and Table 6 for the Vietnamese sample.

**Table 5:** Fifth order partial correlations between scores on the DASS, coping, spiritual bypass, seeking professional help and the NMI controlling for the big five personality domains in the polish sample.

Outcome	NMI Scale			
	Infinitude	Worthiness	Meaning	Total
DASS				
Stress	0.04	−0.14*	0.05	−0.05
Anxiety	0.02	−0.11*	0.02	−0.05
Depression	0.00	−0.28***	−0.01	−0.16**
Total	0.02	−0.20***	0.02	−0.10
Coping				
Problem Focused	0.02	0.21***	0.07	0.15**
Emotion Focused	0.17**	0.02	0.21***	0.17**
Avoidant Coping	0.03	−0.10	0.10	−0.02
Religious Coping	0.35***	−0.15**	0.35***	0.22***
Spiritual Bypass				
Psychological Avoidance	−0.35***	0.08	−0.23***	−0.22***
Spiritualizing	−0.19***	0.03	−0.12*	−0.13*
Help Seeking				
Medical Help	−0.08	0.17***	−0.09	0.03
Psychological Help	−0.04	−0.16**	−0.07	−0.13*

*N* = 328.

*p < 0.05. **p < 0.01. *** *p* < 0.001, two-tailed.

**Table 6:** Fifth order partial correlations between scores on the DASS, coping, spiritual bypass, seeking professional help and the NMI controlling for the big five personality domains in the Vietnamese sample.

Outcome	NMI Scale			
	Infinitude	Worthiness	Meaning	Total
DASS				
Stress	0.05	−0.16**	0.09	−0.06
Anxiety	0.09	−0.13*	0.06	−0.03
Depression	−0.05	−0.27***	0.00	−0.20***
Total	0.03	−0.22***	0.06	−0.11*
Coping				
Problem Focused	0.13*	0.16**	0.06	0.18**
Emotion Focused	0.15**	0.06	0.11*	0.14*
Avoidant Coping	−0.01	−0.05	−0.03	−0.05
Religious Coping	0.25***	0.03	0.30***	0.21***
Spiritual Bypass				
Psychological Avoidance	−0.22***	0.04	−0.33***	−0.16**
Spiritualizing	−0.08	0.02	−0.17**	−0.07
Help Seeking				
Medical Help	−0.06	−0.02	0.00	−0.04
Psychological Help	−0.06	−0.04	−0.11*	−0.09

*N* = 307.

*p < 0.05. **p < 0.01. ***p < 0.001, two-tailed.

With regard to Table 5, there are numerous, expected associations among the variables. Specifically, NMI Worthiness correlated significantly with all aspects of the DASS, supporting previous research that this dimension represents a second, independent pathway to emotional dysphoria. All NMI Scales were significantly related to Religious coping. For Infinitude and Meaning, these associations were positive (*r’s* = 0.35, *p* < 0.001) but for Worthiness the relationship was reversed (*r*[321] = −0.15, *p* < 0.01). Those who score high on Worthiness see less need to rely on their religiousness to manage stress, but higher scores on the other scales are associated with more usage of religion to cope with stress. NMI scale scores on Infinitude and Meaning were also negatively related to Spiritual Bypass, suggesting that more materialistic individuals tend to use religion as a way to avoid dealing with existential issues. An interesting pattern of findings are noted for the Help Seeking scales, where Worthiness was positively associated with medical help seeking (*r*[321] = 0.17, *p* < 0.001) while for psychological help seeking the relationship was negative (*r*[321] = −0.16, *p* < 0.01). Apparently, feeling that one’s life matters and has value is associated with greater help seeking for physical problems, while the lack of worthiness and feelings of despair motivate searching for assistance with mental health issues.

Table 6 presents the same findings for the Vietnamese sample. With the exception of the Help Seeking scales, most of the findings replicate those found in the Polish sample. Worthiness was uniquely and significantly related to all DASS scores. NMI scales, except Worthiness, were related to Religious Coping scores and scores on the Spiritual Bypass scales. Of the 23 significant findings with the Polish sample, 17 (74%) were replicated here.

### 3.5 Causal Modeling linking NMI worthiness to affective dysphoria

Figure 1 presents three different causal models that hypothesize varying causal roles for Worthiness, consistent with Piedmont and Wilkins (2019) ontological model of the Numinous. Model 1 positions Worthiness as an independent predictor of distress from the FFM. Here, Worthiness is hypothesized as its own causal motivation that works additively with personality to impact levels of emotional dysphoria. The remaining models present Worthiness as an outcome of personality, alongside the DASS (Model 2) or an outcome of both personality and the experience of emotional distress (Model 3). Using the SEM software Linear Structural Relations (LISREL version 12.5.1), these different models were examined individually in both the Polish and Vietnamese samples and the results are presented in Table 7.

**Table 7:** Results of the SEM analyses for three models.

Model	𝛘^2^	*df*	𝛘^2^/*df*	RMSEA	SRMR	CFI	IFI	AIC
Polish sample								
1.	32.53	12	2.71	0.07	0.02	0.98	0.98	6603.14
2.	45.60	13	3.51	0.09	0.04	0.97	0.97	6614.21
3.	55.29	17	3.25	0.08	0.05	0.96	0.96	6615.90
Vietnamese sample								
1.	47.95	12	4.00	0.10	0.04	0.95	0.95	5322.51
2.	59.66	13	4.59	0.11	0.05	0.94	0.94	5332.22
3.	67.71	17	3.98	0.10	0.05	0.93	0.93	5332.27

RMSEA, Root Mean Square Error of Approximation; SRMR, Standardized Root Mean Residual; CFI, Comparative Fit Index; IFI, Incremental Fit Index; AIC, Akaike Information Criterion.

As can be seen, Model 1, which hypothesizes Worthiness as a unique causal predictor of distress, is the best fitting model in both the Polish and Vietnamese samples across all fit statistics examined, using criteria proposed by Hu and Bentler (1999) as well as Kline (2016) (i.e., chi square/*df* < 3; RMSEA and SRMR < 0.05; IFI and CFI > 0.95). Of particular interest is the Akaike Information Criterion (AIC). This index is used when non-nested models are being compared. The AIC examines the parsimony and level of fit for each model in terms that can be directly compared. The model with the lowest AIC is seen as the best-fitting model. In Table 7, the AIC analysis indicates that Model 1 fits best. This result does not mean that there is an actual causal effect in the data, but rather the results support our theory that Worthiness is a motivational construct which can directly impact the affective stability of individuals, independently of any personality dispositions, including Emotional Stability. The standardized weights (lambdas) for the six contributing factors in this model were: ES: −0.54 (*t*[12] = −9.23, *p* < 0.001); E: −0.08; O: 0.08; A: −0.05; C: −0.07; W: −0.18 (*t*[12] = −3.62, *p* < 0.001) for the Polish sample. In the Vietnamese sample, similar weights were observed: ES: −0.54 (*t*[12] = −9.91, *p* < 0.001; E: −0.03; O: −0.06; A: −0.03; C: −0.09 (*t*[12] = −3.89, *p* < 0.001; W: −0.17 (*t* [12] = −3.43, *p* < 0.001). In both samples, W made a significant incremental contribution to negative affect *independent* of ES that was about one-third as much as ES. Clearly, W is an important construct to consider in any study examining emotional distress.

## 4 Discussion

This study takes the unusual position of evaluating the cross-cultural generalizability of the NMI in two different samples simultaneously. One sample is a European, Catholic, democratic culture and the other is an Asian, atheistic, communist culture. They are literally and metaphorically on different sides of the world. Yet, as these data have shown, the NMI functions similarly in both contexts. Mean level scores, factor structure, and incremental predictive validities are comparable to United States normative data. Clearly, existential motivations that contribute to human beings’ ultimate sense of personal engagement with life can be meaningfully identified across different cultural contexts. These data provide compelling evidence that supports our contention that the Numinous represents a distinct dimension of personality (Piedmont, 2026).

### 4.1 Potential cultural effects

Yet, some intercultural differences did emerge. First, it has been found in US data that women tend to score higher on the Numinous than men. For this reason, normative data on the NMI are adjusted for both age and gender. In the Polish sample, no gender differences emerged, suggesting that Polish women do indeed score higher than Polish men, but these differences mirror United States differences and are managed well by the adjustments made in standardizing scores with the United States normative data. However, in Chinese samples using the NMI (e.g., Piedmont et al., 2026) as well as in first-generation Vietnamese samples in the United States (Le et al., 2019), men have scored higher than women on all facets of the scale. In the current sample, men scored higher than women only on the total score. On one level this finding is consistent and indicates that the role of men in Asian cultures are more overtly engaged in existential issues than in Western samples. That only one effect was noted may be a consequence of the sample included here. Nonetheless, there is enough data to suggest that there is a real, culturally based, gender effect surrounding the Numinous that is worthy of further investigation.

Another interesting difference between the two samples presented here concerns the factor loadings for two Infinitude items (7 & 8), which evidenced dual loadings on the Meaning dimension in the Vietnamese sample but not in the Polish sample. Piedmont et al. (2026) found similar cross-loadings in their Chinese sample. These items (“I believe that this life is only one stage in a larger, eternal process” and “I think that there are larger forces directing the flow of life”) also appear to resonate with how Meaning is understood by the Vietnamese (a Meaning item is “My religious faith and/or personal philosophy means everything to me”). While Western samples are able to make clean distinctions between these two types of items, in Asian samples there appears to be more of a blurring between the two. This may be a function of the non-Western types of religious and philosophical perspectives that dominate in Asia. Such beliefs may provide less distinctions between Meaning and Infinitude than found in the West. Beliefs related to ancestor worship and family dynamics may lead to aspects of Meaning being indistinguishable from elements of Infinitude. Both require a transpersonal perspective on life. This is another aspect in need of further cultural exploration and development.

A final cultural difference relates to the mean level scores obtained in both samples. It was interesting to note that the more religiously oriented Polish sample scored significantly lower on the NMI scales than the more atheistic/agnostic style characteristic of the Vietnamese. To some degree this slight difference (both groups did score in the “average” range so there is no interpretive differences between the two groups in their scores) may be a function of other cultural differences. Because the NMI has balanced keying, the presence of negatively phrased items may introduce a different response tendency to such items. Wong et al. (2003) have noted that in East Asian samples the use of reverse-scored items negatively impacts the validity of a scale because the respondents may not recognize positively and negatively phrased items as representing similar content. This may negatively impact assessments of reliability, factor structure, and external validity. Attending to these potential cultural issues can ultimately lead to the construction of instruments that are immune to these cultural dynamics, which in turn will lead to more clarity in identifying constructs that are truly universal from more context-dependent dynamics.

### 4.2 Limitations and directions for future research

This report provides a powerful demonstration of the universality of the Numinous by contrasting two very different cultures. The factor structure of the NMI was clearly recovered in both samples and its unique, causal predictiveness replicated US data. Moving forward, there are other issues yet to explore in order to continue to develop the construct of the Numinous. First, in making the claim that the Numinous is an independent personality dimension, to date comparisons were only made using measures of the FFM (e.g., Piedmont et al., 2026). It would be useful to include other taxonomic models of personality, such as the HEXACO model. While the review of the literature did note that religious and spiritual qualities did form a separate, seventh dimension in this model, replicating these results using the NMI would be crucial for demonstrating that ultimate existential concerns are not contained in current personality models. Also, a fuller examination of how the Numinous relates to Openness to Experience is also warranted. To date, correlations between Openness and the Numinous have only used domain-level scores; there may be areas of deeper overlap when specific facets are examined, like Openness to Ideas.

Although the present results support the replicability of the US factor structure, further analyses are needed to determine whether the measure functions comparably across cultural groups. Therefore, future research should extend these findings by examining the cross-cultural equivalence of the NMI through formal measurement invariance testing.

The SEM analyses provided support for the ontological model of the Numinous, which sees Worthiness as a causal predictor of important psychosocial outcomes. While this finding is consistent with other similar research (e.g., Piedmont et al., 2020; Piedmont et al., 2023), the current findings still await the use of experimental methods to confirm these proposed causal relationships in real data.

It would also be helpful to include observer ratings in future research. A singular reliance on self-report data will create spurious associations based on the use of a common method. Demonstrating cross-observer correlations will also demonstrate the importance of the Numinous because it is not a solipsistic aspect of the individual, but a true motivational quality that finds expression in behavior and these expressions can be recognized by others as being existential in nature. This indicates that the Numinous is a construct that is known and shared by individuals; there is a consensual validity to the construct. Longitudinal studies would also be valuable for examining the temporal stability of the Numinous.

One aspect of concern in this study was the lower reliability coefficients found for the Vietnamese version of the IPIP-20 scale in comparison to those obtained in the Polish sample. It was not clear why there was such a difference; it may be because the items were not as clear to respondents in Vietnam as they were to those in Poland, despite the fact that the Big Five personality domains have been well validated across the globe (McCrae, 2001). An important question to answer is whether these lower reliabilities compromise the quality of findings for our Vietnamese sample. While it is a common adage that “reliability sets the upper limit for validity” not all reliability coefficients fit into that relationship. Specifically, alpha reliability is a measure of redundancy among a set of items and does not necessarily have an impact on the validity of scores from such a scale (McCrae et al., 2011). A low alpha means that the items do not overlap, but that does not mean those items do not correlate with external criteria. In looking at the current results, while the Vietnamese IPIP alphas are lower than the Polish version, the pattern of correlations is of equal magnitude in both samples. When comparing the results of the partial correlations in Tables 5 and 6, the magnitudes and patterns were very similar. In fact, of the 23 significant associations in Table 5 (the Polish Sample), 17 of them (74%) were replicated in the Vietnamese sample (see Table 6). In examining the SEM results, where personality is also used as a covariate, a similar pattern of findings was also obtained. Such consistency in effects would be unlikely if the lowered reliability was adversely affecting the findings. We are confident in our findings here because in a different sample of mainland Chinese subjects, we used a longer version of the IPIP (the 50-item scale), which had higher alpha reliabilities than found with our Vietnamese sample and found a very similar pattern of partial correlations with the DASS (the only overlapping scale; Piedmont et al., 2026). Given the consistency of findings both in this study and in others, we feel that the lower alpha reliabilities for the IPIP in the Vietnamese sample were not creating any problems with the data. Nonetheless, more attention needs to be given to understanding the IPIP-20 in this culture.

Finally, future research can also include direct measures of self-esteem, meaning in life, and purpose in life to assess the construct validity of the NMI. Sensitivity analyses should then be conducted to determine whether the relationships between the NMI and these constructs remain robust after excluding potentially overlapping DASS items.

### 4.3 Conclusion

This study is another step forward in the empirical documentation of the universal value of existential motivations and their utility for understanding human behavior. Most importantly, if the Numinous is indeed a separate dimension of personality, then the field will need to realign its understanding of personality and its impact on behavior. The Numinous is seen as a unique human trait that is at the core of our adaptiveness and evolutionary success as a species (Piedmont, 2026). Its systematic inclusion for understanding people will extend the field’s ability to better understand psychological problems currently refractory to treatment (e.g., Moral Injury, Body Image Dysphoria, Suicide) as well as suggest new treatment techniques that may have the potential to create truly transformative therapeutic experiences.

## Data Availability

The data supporting the conclusions of this article can be made available upon request to the corresponding author.
